# Prior knowledge and perceived impacts of tick-borne disease education among indigenous bison workers at an annual roundtable training

**DOI:** 10.3389/fpubh.2025.1738923

**Published:** 2026-01-21

**Authors:** Alexandrea M. Welch, Mikiya Reuther, Louise I. Lynch-O’Brien, Mystera M. Samuelson, Shaun T. Cross

**Affiliations:** 1 Department of Environmental, Agricultural, and Occupational Health, College of Public Health, University of Nebraska Medical Center, Omaha, NE, United States; 2 Central States Center for Agricultural Safety and Health, College of Public Health, University of Nebraska Medical Center, Omaha, NE, United States; 3 InterTribal Buffalo Council, Rapid City, SD, United States; 4 Department of Entomology, Institute of Agriculture and Natural Resources, University of Nebraska-Lincoln, Lincoln, NE, United States; 5 Nebraska One Health, Institute of Agriculture and Natural Resources, University of Nebraska-Lincoln, Lincoln, NE, United States

**Keywords:** agricultural and occupational health, bison, education, indigenous, tick-borne diseases

## Abstract

Ticks are important vectors of human and animal disease, and outdoor workers are at elevated risk of tickborne diseases (TBDs). This risk is particularly relevant for Indigenous herd workers managing bison (colloquial: buffalo), which are culturally, spiritually, and economically significant. To address this gap, the InterTribal Buffalo Council and Central States Center for Agricultural Safety and Health partnered with Tick Tag Go (University of Nebraska–Lincoln) to provide education on tick identification, prevention, and TBDs at the 2024 Bison Worker Safety & Herd Health Roundtable. Thirty-four participants, including herd managers, workers, and tribal leaders, engaged in the session, with 27 completing a post-presentation survey. The presentation was well received: 96.3% reported gaining new knowledge, 70.4% indicated they were more likely to implement preventive practices, and 74.1% found the material relevant to their needs. Nearly half (44.4%) expressed interest in additional community-based programming. To our knowledge, this is the first report assessing perceptions of ticks and TBDs among Indigenous agricultural workers. Findings highlight both knowledge gaps and receptiveness to education, underscoring the importance of culturally tailored outreach to reduce TBD risk in Indigenous communities.

## 1 Introduction

Ticks are vectors for various pathogens, which pose significant public health risks. There are continuing increases in the number of reported tickborne diseases (TBD) each year and TBDs now account for over 75% of vector-borne infections in the United States (1). Cases of tick-borne diseases have more than doubled in recent years, yet it is suspected that this is largely underestimated as many TBD cases go unreported (1, 2). Although there are multiple risk factors for acquiring a TBD, outdoor workers (e.g., agriculture workers) are at an increased risk for TBD due to the time spent in tick habitats (3–5).

Bison (hereto referred to as buffalo as it is a colloquial and sometimes the preferred name amongst Tribes) are significant to the culture and lifestyle of Indigenous people in the United States. In the 1800s, over 60 million buffalo were slaughtered and nearly brought to extinction, leaving only a fraction of the original population (6). The InterTribal Buffalo Council (ITBC), in a remarkable collaboration with Tribes, is working tirelessly to return buffalo to reservations across the country (6, 7). Buffalo pastures sit in habitat that is often optimal for ticks, including along the edges of forest vegetation and long grasses. Therefore, Indigenous herd workers can often find themselves in these habitats by nature of their work.

There is a notable research gap regarding the relationship between ticks, Indigenous agricultural workers, and buffalo. One challenge includes the underrepresentation of the Native American population in epidemiological studies and scientific data (8). Native American populations tend to live in more rural areas that may more frequently expose them to ticks and epidemiological surveillance suggests increased burden of TBDs in these communities (9–12). Every year, the Central States Center for Agricultural Safety and Health (CS-CASH), in collaboration with the ITBC, hosts a Bison Worker Safety & Herd Health Roundtable. This effort aims to educate Indigenous community members and herd workers about occupational hazards and threats to their workers and herds. During the 2024 curriculum, interest was expressed in learning more about ticks and TBDs. This working relationship between CS-CASH, ITBC, and Indigenous communities provided a unique avenue for us to educate and empower attendees regarding perceptions and practices surrounding tickborne disease, ticks, preventative practices, and misinformation.

To capture generalizable reception of the material in this focus group, we designed a mixture of questions that were presented both during and after the presentation. These questions were designed to capture the background knowledge regarding ticks and TBDs as well as understand perceived impacts and willingness to implement preventative practices. The feedback from this focus group and information session allows us to refine and adapt our messaging to maximize its impact on other Indigenous communities in the future. The results of this intervention will contribute significantly to furthering the unmet needs of empowering Indigenous communities regarding TBDs.

## 2 Materials and methods

### 2.1 Focus group participants

The study participants attended the 2024 Bison Worker Safety & Herd Health Roundtable hosted by the Central States Center for Agricultural Safety and Health (CS-CASH) and the InterTribal Buffalo Council (ITBC). The meeting occurred on July 24–25, 2024, at the Winnavegas Casino Resort in Sloane, Iowa. The participants included bison workers, herd managers, Indigenous community leaders, wildlife biologists, health professionals, and researchers. Representatives from all Tribal Nations who have or hope to have buffalo restored to their lands are invited through collaborations with the ITBC. Thus, this information serves as a preliminary assessment of knowledge and attitudes regarding ticks and tick-borne diseases.

This research was deemed as non-Human Subjects Research by the University of Nebraska Medical Center Institutional Review Board Committee. No personally identifiable information was collected, and the purpose of these responses were for programmatic evaluation.

### 2.2 Educational material

The Nebraska One Health system supports integrated training, research, extension, and outreach opportunities. Hosted under this organizational structure is Tick Tag Go, led by Dr. Louise Lynch-O’Brien, and is focused on educating the public on the impacts of ticks and TBDs. Tick Tag Go has a diverse variety of resources for educational outreach (13). In conjunction with Tick Tag Go, we selected, crafted, and disseminated educational materials to present and share with attendees using culturally appropriate language and targeting areas of concern expressed by Tribal herd workers over the last 6 years of collaborative work with these communities. Educational material was built around perceptions, knowledge, and preventative practices for TBDs.

### 2.3 Feedback and participant questionnaires

In-presentation questions were formulated to prompt participants to raise their hands and/or provide verbal responses. The questions were both asked verbally and written on the corresponding presentation slide. Total counts and verbal responses were recorded.

Following the presentation, a short, post-presentation questionnaire was administered to gauge the impact of the presentation material, understand how behaviors regarding tickborne diseases may change following the presentation, and identify areas of improvement for future educational outreach events. This questionnaire consisted of 11 questions. Eight of the 11 questions were Likert scale questions where participants reported if they “agree,” are “neutral,” or “disagree.” The remaining three questions were short answer responses.

Questions were required to adhere to non-Human Subject Research IRB requirements focused on the scope of programmatic evaluation. However we aligned, where possible, with questions that assess knowledge, behavior, and traditional knowledge regarding vector-borne diseases in Indigenous communities. This first step in assessing perceptions will guide future interventions aimed at improving health outcomes for Indigenous people in North America. Further, this is the first step in developing a comprehensive approach to OneHealth initiatives that are culturally appropriate and both needed as well as requested by the community, increasing the utility of the training and discussion (14–18).

Descriptive analysis was performed based on the participant’s perceptions of ticks, tickborne diseases, and preventative measures. The data was presented as numbers and percentages for the categorical data.

## 3 Results

There were 34 total attendees at the Bison Worker Safety & Herd Health Roundtable, comprised of affiliates with Indigenous working groups (e.g., ITBC, herd managers, etc), that participated in the presentation. Of the initial 34 attendees, 27 participants were available to complete a survey following the presentation for a 79.4% response rate. The presentation was well received by participants, with 55.56% of participants answering in the final Roundtable event survey that the tick education presentation was the most useful. The presentation and survey highlighted three significant themes: tick identification and ecology, prevention, and tickborne diseases. Questions and responses that were presented during the course of the presentation are located in Table 1 while the post-presentation evaluation data are located in Table 2.

**Table 1:** List of questions and their responses that were provided during the course of the presentation.

In-presentation questions		
Question	No	Yes
Have you ever found a tick on yourself?	2 (5.9%)	32 (94.1%)
How many of you work in a habitat that could be described like one of these pictures? (images of various types of tick habitat were shown)	3 (8.8%)	31 (91.2%)
How many of you have heard ticks can jump or fall from trees?	29 (85.3%)	5 (14.7%)
Do all ticks transmit Lyme disease?	33 (97.1%)	1(2.9%)
Can animals get tick-borne diseases?	0 (0%)	34 (100%)
How many of you are performing tick checks after working outside?	23 (67.6%)	11 (32.4%)
How many of you are familiar with alpha-gal syndrome and the allergy it causes?	18 (53%)	16 (47%)

A total of 37 participants attended the presentation.

**Table 2:** Post-presentation questionnaire provided Likert scale questions to gauge reception, impact, and likelihood to implement these educational materials.

Survey responses				
Question	Disagree	Neutral	Agree	N/A
This presentation provided me with new knowledge of ticks and tick-borne diseases	0 (0%)	1 (3.7%)	26 (96.3%)	0 (0%)
This presentation has made me confident in identifying where ticks may be found on my body	0 (0%)	5 (18.5%)	22 (81.5%)	0 (0%)
I feel confident about identifying different species of ticks with the resource materials given.	0 (0%)	6 (22.2%)	21 (77.8%)	0 (0%)
I am now more likely to implement preventative practices for tick bites following this program. (tick checks, repellents, etc.)	1 (3.7%)	6 (22.2%)	19 (70.4%)	1 (3.7%)
This presentation provided me with adequate resources to find information on tick-borne diseases.	0 (0%)	2 (7.4%)	25 (92.6%)	0 (0%)
The presentation materials were understandable.	1 (3.7%)	1 (3.7%)	25 (92.6%)	0 (0%)
I have an interest in having individualized programs in my own communities.	2 (7.4%)	13 (48.1%)	12 (44.4%)	0 (0%)
The presentation content was relevant to my needs.	0 (0%)	7 (25.9%)	20 (74.1%)	0 (0%)

A total of 27 of the original 34 participants remained available to complete the questionnaire.

### 3.1 Theme 1: tick identification and ecology

Participants were asked about their immediate thoughts and perceptions surrounding ticks entering into the presentation. Responses included they believe ticks to be “creepy,” “disgusting,” and “not sure why they exist.” Consistent with their occupations as predominantly outdoor, agricultural workers, almost all participants (*n* = 31, 91.2%; Table 1) reported working in a wooded area, tall grass, or pasture consistent with tick habitat (19). Nearly all the participants (*n* = 32, 94.1%; Table 1) reported that they have found a tick on themselves. Participants were informed where ticks are found in these habitats and how they seek for hosts, during which five participants (14.7%; Table 1) reported they were taught the misconception that ticks jump or fall off trees to find a new host.

The participants were shown a picture of four tick “look-alikes” and one tick. When asked to identify a picture of a tick, 88% (*n* = 30; Table 1) identified the tick correctly. Two participants (5%) incorrectly identified a bed bug as a tick and two participants (5%) incorrectly identified an ant as a tick. The other two pictures shown were a louse and a beetle, and no participants incorrectly selected those options. The group knew different types of ticks existed but expressed that they knew little about the individual types. In one of the open-ended responses following the presentation, one participant suggested “more identification information on the most common species.” However, a majority of participants (77.8%; Table 2) did state they felt more confident about identifying different species of ticks with the resource materials given.

### 3.2 Theme 2: prevention

Although identification and knowledge regarding what environments ticks may be found is crucial, active implementation of best practices to avoid tick bites is key to preventing TBDs (20). Participants provided responses on which preventative measures were currently being used to prevent tick bites. The most common answer was “bug spray.” Several participants stated they were tucking pants into socks and shirts into pants. One participant stated that they were using garlic as a tick prevention method, which has been shown to be a deterrent, albeit weak (21). Besides preventative practices when entering or working in tick habitat, participants were surveyed for their use of tick checks on their bodies after returning from the field. Only approximately a third of participants (*n* = 11, 32.4%; Table 1) reported doing tick checks. Of these participants, three (11.1%) reported doing tick checks for 5–10 min, and two (7.4%) reported checking for 10-plus minutes.

### 3.3 Theme 3: tick-borne diseases

Of the 34 respondents, 33 (97%; Table 1) had previously learned or heard about Lyme disease. Curious about their knowledge of emerging, non-infectious TBDs, we surveyed regarding Alpha-gal syndrome or sometimes called the “red meat allergy.” About half of the respondents (*n* = 16, 47.1%; Table 1) were familiar with Alpha-gal syndrome, consistent with other survey reports on this emerging tick-associated allergy, including within physicians (22). All participants (100%; Table 1) knew that both animals and humans are susceptible to TBDs. When surveyed about the capacity of ticks to transmit diseases, several participants correctly noted verbally to the group that not all ticks are responsible for Lyme disease or other TBDs. Additional resources were provided (e.g., Center for Disease Control and Prevention websites) for continued learning. 92.6% of participants agreed that these materials provided them with adequate resources to find information on tick-borne diseases (Table 2).

This theme of the presentation engaged the most participation. Participants asked for additional details regarding TBDs and whether ticks can serve as vectors for other diseases. For example, one participant asked if ticks can transmit the infectious prions that cause Chronic Wasting Disease (CWD). Additional follow-up questions regarding incubation periods, symptoms, and treatments for the different TBDs were asked. Additional interest in our line of research was also observed with participants inquiring if we have contracted Lyme disease due to our research with ticks.

### 3.4 Perceived reception and implementation of preventative practices

To identify the perceived reception/impact of these materials, we administered a post-presentation questionnaire which 27/34 (79.4%) participants completed. Generally, the majority of participants responded “agree” for each of the questions asked (Table 2). After the presentation, the respondents responded in the short answer section that they felt: interested, helped, informed, aware, armed, more cautious, not scared anymore, and confident.

Additionally, over the course of the two-day training event, we received additional colloquial feedback. We have historically found with this community that informal feedback can be preferred rather than through structured surveys. Participants provided statements such as “This was extremely helpful and I want to know if there is more I can do,” “This aligns well with our goals to educate and be aware regarding diseases workers may get,” and “Please contact me further and find a way to talk to the rest of my tribe.” These statements align with the survey results that the presentation content was relevant to their needs (*n* = 20, 70.4%) and an interest in having individualized programs in their communities (*n* = 12, 44.4%) (Table 2).

Participants agreed that they would be more likely to implement preventative practices (n = 19, 70.4%; Table 2). On the second day of the Roundtable event, participants traveled to a buffalo pasture. Several participants brought tick removal kits that we provided, some asked if certain “bugs” found on themselves were ticks, and one participant did identify and remove a tick from themself while in the field after performing a tick check.

## 4 Discussion

In partnership with ITBC, CS-CASH participated in the annual Bison Worker Safety & Herd Health Roundtable, we were able to engage Indigenous herd workers, agricultural staff, and Tribal leaders in a discussion on ticks and tickborne diseases (TBDs). This study addresses an important gap in knowledge, as few studies have specifically assessed agricultural workers’ perceptions of TBDs, and even fewer have focused on Indigenous communities. Despite evidence of increasing TBD burden in the Indigenous populations (9–11), they remain underrepresented in research. Here, we captured both perceptions and reception of tailored educational outreach, with over half of participants reporting (55.6%) that the tick education presentation was the most useful session at the event. Providing such education in trusted setting likely contributed to such a positive reception for rural workers (23). Here we present both existing knowledge regarding ticks and TBDs in Indigenous communities, which can be leveraged to ensure that future initiatives address the concerns of the community. These insights can be leveraged to address teaching inequity in vector-borne disease management within these at risk, but underrepresented, populations (24).

Three dominant themes emerged: tick identification and ecology, prevention practices, and TBD awareness. While most participants reported prior exposure to ticks (94.1%), gaps and misconceptions were uncovered, including 14.7% reporting the belief that ticks fall or jump from trees to locate a host. Most participants expressed increased confidence in recognizing ticks (77.8%) and a stronger knowledge in ticks and tick-borne diseases (96.3%) after the presentation, suggesting that educational materials had an immediate impact. Preventative practices, however, remained inconsistent, with fewer than one-third of participants regularly conducting tick checks (32.4%) despite reporting frequent exposure to tick habitats (91.2%). This disconnect highlights the importance of reinforcing prevention strategies (20, 25) and ensuring they are performed within agricultural work routines. Importantly, interest extended beyond Lyme disease, as nearly half of participants (47%) were already familiar with emerging conditions such as Alpha-gal syndrome and sought further information on other TBDs. With the materials and resources provided here, 92.6% reported they felt adequate resources to find information on TBDs. Participants also requested more species-specific tick identification resources and community-tailored educational programming, underscoring both the receptiveness of the audience and the potential for long-term impact when outreach is culturally relevant and participatory.

This was a preliminary investigation with several limitations, including its small sample size, reliance on self-reported qualitative data, and restriction to a single event. Additionally, hand-raising responses during the presentation may introduce bias (26). Nonetheless, participants represented multiple Tribes across the greater Midwest, and the strong reception suggests that expanding this programming has potential for broader impact. With 96.3% of participants reporting they acquired new knowledge, 74.1% agreeing the material was relevant to their needs, and 44% requesting additional programs, this study demonstrates the need and desire for increased tick and TBD education in Indigenous agricultural communities. Moving forward, continued education and outreach will be critical to addressing TBD risk at the intersection of Indigenous workers, buffalo herds, and tick ecology.

## Data Availability

The original contributions presented in the study are included in the article/supplementary material, further inquiries can be directed to the corresponding author.
